# Supplementary material for: Insights from human NF-κB knockouts
Source: EMBO Rep. 2025 Jun 18;26(14):3491–505. doi: 10.1038/s44319-025-00500-x (PMC12287409; doi:10.1038/s44319-025-00500-x)
Supplement: Supplementary file 1 — Appendix [file 44319_2025_500_MOESM1_ESM.pdf]

Appendix

Appendix Figure S1 2

Appendix Figure S2 3

Appendix Figure S3 4

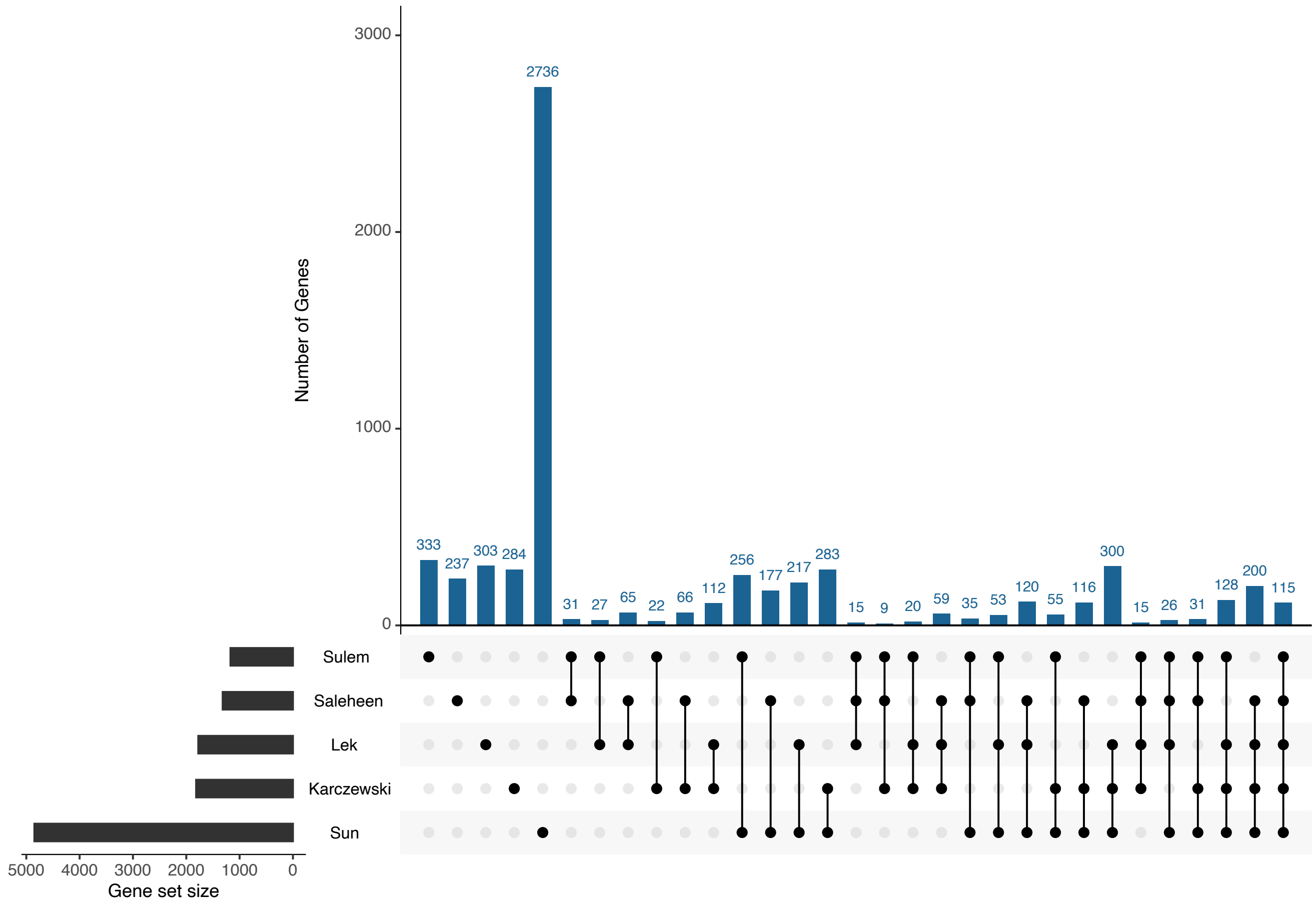

Pfisterer et al, Appendix Figure S1.
Comparative analysis of pLOF human genes identified in different sequencing studies. The UpSet plot shows shared genes between indicated gene sets. The plots were generated with the R package UpSetR version 1.4.0.

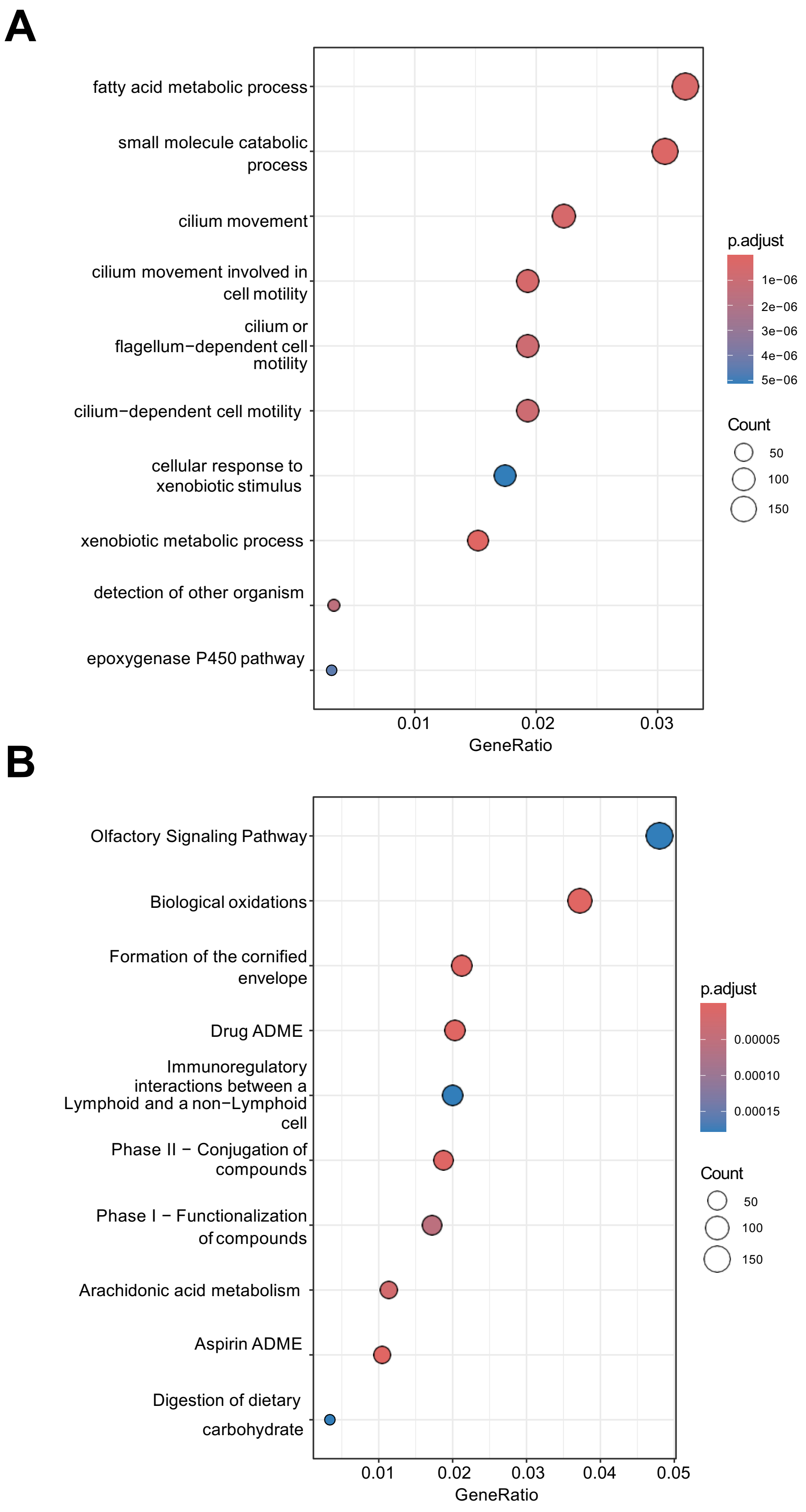

Pfisterer et al, Appendix Figure S2.
Over-representation analysis of non-essential genes. The 6,689 non-essential human genes were analyzed by overrepresentation analysis using GO biological process (A), and Reactome (B) as the reference datasets. Significance of enrichment is indicated by Benjamini-Hochberg adjusted P values and bar colors.

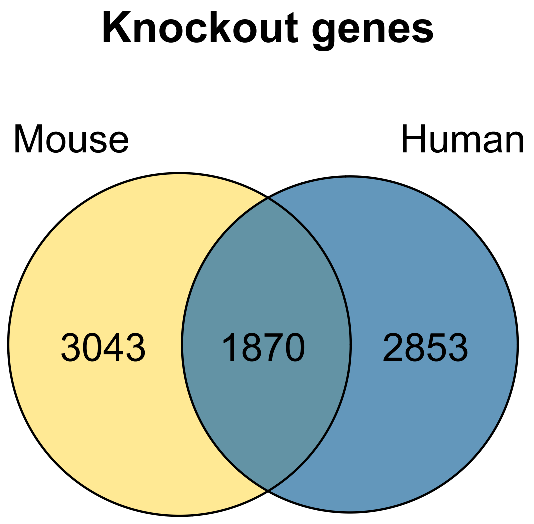

Pfisterer et al, Appendix Figure S3
Comparison of non-essential mouse and human genes. The non-essential human gene list compiled here (6,689 genes) and non-essential mouse genes (5,820 genes), as defined as viable knockouts from The International Mouse Phenotyping Consortium (Cacheiro et al, 2020) were analysed. Only murine genes with a single ortholog in the human genome were included in the analysis.
